# Potential of quantitative *SEPT9* and *SHOX2* methylation in plasmatic circulating cell-free DNA as auxiliary staging parameter in colorectal cancer: a prospective observational cohort study

**DOI:** 10.1038/s41416-018-0035-8

**Published:** 2018-04-03

**Authors:** Julia Bergheim, Alexander Semaan, Heidrun Gevensleben, Susanne Groening, Andreas Knoblich, Jörn Dietrich, Julia Weber, Jörg C. Kalff, Friedrich Bootz, Glen Kristiansen, Dimo Dietrich

**Affiliations:** 1 Department of Otolaryngology, Head and Neck Surgery, University Hospital Bonn, Bonn, Germany; 2 Department of General, Visceral, Thoracic and Vascular Surgery, University Hospital Bonn, Bonn, Germany; 3 Institute of Pathology, University Hospital Bonn, Bonn, Germany; 4 Department of Visceral Surgery, Marien-Hospital Bonn, Bonn, Germany

**Keywords:** Diagnostic markers, Colorectal cancer

## Abstract

**Background:**

Septin 9 (*SEPT9*) and short stature homeobox 2 (*SHOX2*) methylation in circulating cell-free DNA (ccfDNA) are powerful biomarkers for colorectal cancer (CRC) screening, as well as head and neck squamous cell carcinoma staging and monitoring. In the present study, we investigated *SEPT9* and *SHOX2* ccfDNA methylation as auxiliary pre and post-therapeutic staging parameters in CRC patients.

**Methods:**

ccfDNA methylation was quantified in 184 prospectively enrolled patients prior to and 3–10 days after surgery, and biomarker levels were associated with clinico-pathological parameters.

**Results:**

Pre-therapeutic levels of *SHOX2* and *SEPT9* ccfDNA methylation were strongly associated with Union for International Cancer Control (UICC) stages, tumour (T), nodal (N), and metastasis (M) categories, and histological grade (all *P* ≤ 0.001), as well as lymphatic invasion and extracapsular lymph node extension (all *P*< 0.05). Post-therapeutic *SHOX2* and *SEPT9* ccfDNA methylation levels correlated with UICC stage (all *P*  <0.01). *SEPT9* ccfDNA methylation further allowed for an accurate pre- and post-therapeutic detection of distant metastases (AUC_pre-therapeutic_ = 0.79 (95%CI 0.69–0.89), AUC_post-therapeutic_ = 0.93 (95% CI 0.79–1.0)).

**Conclusions:**

DNA methylation analysis in plasma is a powerful pre and post-therapeutic diagnostic tool for CRC and may add valuable information to current TNM staging, thereby holding the potential to assist in the development of individually tailored treatment protocols.

## Introduction

Despite dramatic reductions in overall incidence and mortality, colorectal cancer (CRC) remains the third most commonly diagnosed cancer in both men and women in the United States.^1^ Current treatment algorithms are based on three pillars: surgery, (radio-)chemotherapy, and targeted therapy.^2^ The clinical management of CRC is mainly determined by the Union for International Cancer Control (UICC)/TNM stage and distinct genetic biomarkers (e.g., mismatch repair proteins or epidermal growth factor receptor (EGFR) status).^3^ The mainstay of curative therapy for stage I/II CRC is surgical resection; however, there is an ongoing debate as to whether adjuvant chemotherapy may be beneficial for a subgroup of stage II patients with high-risk features.^4,5^ Although clinical staging and, as a consequence, treatment decisions are predominantly guided by radiologic imaging,^6^ the ability of up-to-date imaging modalities to identify systemic tumour burden is still far from optimal. Up to 25% of liver metastases smaller than 10 mm, for instance, might be not be detected,^7^ and patients would accordingly be significantly undertreated. A validated blood-based biomarker for CRC may help to identify patients with radiologically undetectable (micro-)metastases, who would benefit from neoadjuvant therapy or intensified treatment algorithms.^8^

For CRC patients with synchronous resectable metastases, the National Comprehensive Cancer Network (NCCN) currently suggests neoadjuvant therapy followed by operative and adjuvant treatment.^9^ Nonetheless, long-term benefits of neoadjuvant treatment has to been weighed against an increased perioperative morbidity and the limitation for adjuvant chemotherapy in case of recurrence.^10^ Post-therapeutic detection of (occult) metastases or residual disease might allow for an early initiation of a palliative treatment. Blood-based biomarkers that might further assist in treatment decisions are therefore urgently needed.

Attributable to its stability and cancer specific alteration, DNA methylation has emerged as a promising source for tumour biomarkers. Moreover, tumour-derived circulating cell-free DNA (ccfDNA) with epigenetic aberrations can be reliably assessed against a background of non-tumourous ccfDNA with high precision; thereby adding valuable information on prognosis, diagnosis, and putative response to treatment.^11,12^ Promoter hypermethylation of septin 9 (*SEPT9*) has previously been confirmed as a potent biomarker in various cancers including CRC and its precursor lesions.^13–18^ As a consequence, *SEPT9* methylation in ccfDNA has recently received approval by the U.S. Food and Drug Administration (FDA) as first blood-based CRC screening test. In accordance, promoter methylation of short stature homeobox 2 (*SHOX2*) has shown excellent results in screening and diagnosis of lung cancer patients.^19–21^ Quantitative *SHOX2* and *SEPT9* methylation levels have been successfully applied for the diagnosis of colonic adenomas,^16^ the detection of malignant cells in pleural effusions and ascites,^22,23^ and very recently, for the diagnosis, prognosis, and molecular staging of head and neck squamous cell carcinomas (HNSCC).^15^ In the latter study, methylation levels of both biomarkers were significantly associated with nodal (N) and tumour (T) categories as well as histopathologic grade.^15^ In addition, tumour recurrence and the diagnosis of a second malignancy were detected almost one year prior to clinical or radiologic appearance and provided a strong prognostic biomarker which was independent of TNM. Methylation testing in HNSCC proved to be a valid and extremely powerful diagnostic tool for molecular disease staging, risk stratification, and disease monitoring and, once established in clinical routine, might positively influence the outcome of many patients.

The present study prospectively explores the value of quantitative *SEPT9* and *SHOX2* methylation levels in ccfDNA for disease staging of CRC patients in addition to current TNM staging system and along with the established serum biomarkers carcinoembryonic antigen (CEA) and carbohydrate antigen 19–9 (CA 19–9).

## Patients and Methods

### Patients and study design

#### Patients

A total of 184 CRC patients treated at the Departments of Visceral Surgery at the University Hospital of Bonn and the Marien-Hospital Bonn (Germany) between November 2013 and December 2016 were prospectively enrolled in the present study. In addition, 395 primary colorectal adenocarcinomas and 45 normal adjacent tissues from The Cancer Genome Atlas (TCGA) Research Network (http://cancergenome.nih.gov/.) were included and analysed retrospectively.

#### Inclusion criteria

All patients presented with histologically confirmed primary adenocarcinoma of the colorectum. All prospectively enrolled patients had a history free of a second malignancy of at least 3 years. Blood samples were taken prior to (pre-therapeutic samples) and 3–10 days after surgery (post-therapeutic samples) except for neoadjuvantly treated patients from whom pre-therapeutic samples were taken prior to neoadjuvant treatment. Supplemental Fig. 1 shows a CONSORT diagram of the enrollment strategy and available biomarker results of the prospective study arm. The study protocol was approved by the ethics committee of the University Hospital Bonn (vote no. 222/13). All patients had provided written informed consent.

### Sample preparation and *SEPT9* and *SHOX2* methylation quantification

EDTA-stabilised blood plasma (3 mL) was prepared, and quantitative DNA methylation analysis of ccfDNA was performed as described in detail earlier.^15^ Plasma was prepared within 8 h after blood sampling in order to ensure sample stability.^24^ Patients’ samples were classified as ccfDNA methylation-positive using previously validated cut-offs (*SHOX2*: 0.25%, *SEPT9*: 0.075%).^15^

Methylation results obtained from the TCGA Research Network were generated using the Infinium HumanMethylation450 BeadChip (Illumina, Inc., San Diego, CA, USA). M-values from the TCGA Colon and Rectal Cancer (COAD/READ=CRAD) cohort were downloaded from the UCSC Xena browser (http://xena.ucsc.edu) and analysed. The two beads cg12783819 and cg12993163, that hybridise to CpG-sites within the target region of the *SEPT9* and *SHOX2* qPCR assays, were evaluated.^15^

### CEA and CA 19-9 quantification

CEA and CA 19–9 serum levels were determined using ADVIA Centaur CEA and ADVIA Centaur CA 19–9 tests (Siemens Healthineers GmbH, Erlangen, Germany). Serum testing was performed by SYNLAB laboratories (SYNLAB International GmbH, Munich, Germany). Positivity was defined using broadly accepted cut-offs (CEA: 5 ng/mL, CA 19–9: 37 U/mL).^25–27^ For statistical analyses, biomarker levels below the lower limits of quantification reported as ≤0.5 ng/mL (CEA) and ≤1.2 U/mL (CA 19–9) were set to 0.5 ng/mL and 1.2 U/mL, respectively.

### Statistical analyses

Kruskal–Wallis tests, Spearman’s rank correlations, paired *t* tests, and Wilcoxon–Mann–Whitney *U* tests were performed to analyse biomarker levels. Median methylation levels were reported including Interquartile Ranges (IQR). Two-sided *P*-values <0.05 were considered statistically significant. The Area Under the Curve (AUC) of the Receiver Operating Characteristic (ROC) was computed as a measure of test diagnostic accuracy. AUCs were reported including 95% confidence intervals (95% CIs).

## Results

### *SHOX2* and *SEPT9* DNA methylation in CRC tissue

**Fig. 1:**
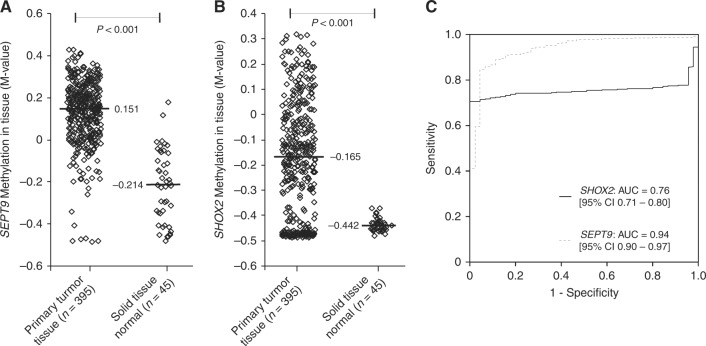
*SEPT9* and *SHOX2* DNA methylation levels in colorectal cancer and normal tissue. *SEPT9* (**a**) and *SHOX2* (**b**) methylation levels in primary colorectal tumours (*n* = 395) and normal solid tissue (*n* = 45). Each rhombus reflects one sample measurement. Median values are given (black bars). *P*-values refer to Mann–Whitney *U* test. Receiver Operating Characteristic (ROC) of *SEPT9* and *SHOX2* methylation for the discrimination between colorectal cancer and normal tissues (**c**)

Methylation levels of 395 primary CRC and 45 solid normal adjacent tissues from the TCGA Research Network were analysed. *SHOX2* and *SEPT9* were found to be hypermethylated in tumour tissues compared to normal adjacent tissues (diagnostic accuracy: AUC_*SEPT9*_ = 0.94, 95% CI [0.90–0.97], AUC_*SHOX2*_ = 0.76, 95% CI [0.71–0.80], *P* < 0.001; Fig. 1). Of note, a group of CRC tissue samples exhibited *SHOX2* methylation levels below those of normal adjacent tissues (Fig. 1) leading to a significantly lower AUC compared to *SEPT9*.

### *SHOX2* and *SEPT9* ccfDNA methylation in plasma for molecular staging prior to treatment

**Fig. 2:**
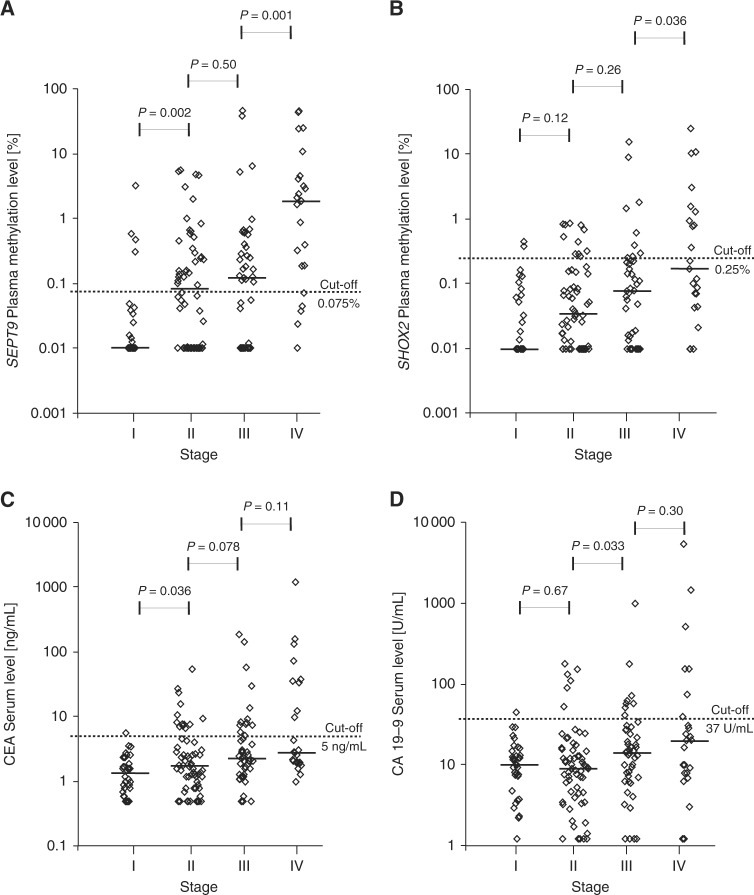
Stage-dependent pre-therapeutic biomarker levels. *SEPT9* (**a**) and *SHOX2* (**b**) ccfDNA methylation in plasma of stage I (*n* = 29), II (*n* = 58), III (*n* = 43), and IV (*n* = 23) colorectal cancer patients prior to surgery. CEA (**c**) and CA 19–9 (**d**) serum levels of stage I (CEA: *n* = 36; CA 19–9: *n* = 35), II (*n* = 62), III (*n* = 47), and IV (*n* = 24) patients before surgery. *P*-values refer to Wilcoxon Mann-Whitney *U* tests. Methylation levels below 0.01% were set to 0.01% in order to allow for a logarithmic illustration

A total of 184 CRC patients were prospectively enrolled in our study cohort. Patients’ characteristic and clinico-pathological features are described in detail in Tables 1 and 2. Furthermore, detailed patient and sample-specific clinico-pathologic parameters and biomarker levels are summarised in Supplemental Table 1. Pre-therapeutic *SEPT9* and *SHOX2* ccfDNA methylation levels were available for 155 out of 184 patients (84.2%). Quantitative methylation levels prior to surgery were significantly associated with UICC stage, TNM categories, histological grade, extracapsular lymph node extension, and lymphatic invasion (all *P* < 0.05) but not with tumour localisation and vascular invasion (all *P* > 0.05, Tables 2 and 3). Most interestingly, ccfDNA methylation levels of *SEPT9* were stage-dependent and showed a stepwise increase in UICC-stages (I-IV), local tumour stages (T_1_–T_4_), nodal status (N_0_-N_2_), histopathologic grades (G_1_–G_3_), and lymphatic invasion (L_0_–L_1_). A significant difference of *SEPT9* methylation levels was further demonstrated between local tumour and systemic tumour burden (M_0_ vs. M_1__a_) but not between one and multiple metastatic sites (M_1__a_ vs. M_1__b_, Tables 2 and 3). While *SEPT9* methylation levels revealed a significant increase from UICC stage I to II (*P* = 0.002) and stage III to IV (*P* = 0.001), no significant difference between stage II and III was detected (*P* = 0.50, Fig. 2a).

**Table 1:** Patients’ characteristics

	Patient number
All patients	184 (100%)
Age	
≤50 years	12 (6.5%)
51–60 years	33 (17.9%)
>60 years	139 (75.5%)
Median age (years)	71
Mean age (years)	69.3
Age range (years)	26–90
Gender	
Female	86 (46.7%)
Male	98 (53.3%)
Smoking and drinking habits	
Non-smokers	134 (72.8%)
Smokers (current and former)	46 (25.0%)
Unknown smoking status	4 (2.2%)
Range pack/years	0–80
Median pack/years (smokers only)	0
Mean pack/years (smokers only)	7.9
Non-alcoholic	158 (85.9%)
Alcoholic	22 (12.0%)
Unknown alcohol consumption	4 (2.2%)
Pre-existing conditions	
None	19 (10.3%)
Diseases of cardiovascular system	111 (60.3%)
Diseases of respiratory system	19 (10.3%)
Diseases of metabolism or endocrinological system	78 (42.4%)
Diseases of kidney and urinary tract	10 (5.4%)
Diseases of hepatic and biliary system	35 (19.0%)
Pancreatic diseases	3 (1.6%)
Neurological and psychiatric diseases	19 (10.3%)
Haematological diseases	4 (2.2%)
Rheumatologic diseases	4 (2.2%)
Skin diseases	2 (1.1%)
Skeletal diseases	10 (5.4%)
Ophthalmologic diseases	4 (2.2%)
Infectious diseases	4 (2.2%)
Diseases of genital tract	19 (10.3%)
Colon adenoma	32 (17.4%)
Colon polyp	23 (12.5%)
Colon and sigma diverticulosis	37 (20.1%)
Inflammatory colon diseases	1 (0.5%)
FAP or HNPCC	1 (0.5%)
Reflux, Barrett’s oesophagus, gastrointestinal ulcers	30 (16.3%)
Anorectal diseases	8 (4.3%)
Status after other malignant tumours^a^	18 (9.8%)
Status after other benign tumours	3 (1.6%)

Characteristics of the CRC patient cohort (184 patients). First-line treatment of CRC patients consisted of surgery in 56% (103/184), surgery and adjuvant radio-chemotherapy in 2% (4/184), surgery and adjuvant chemotherapy in 31% (57/184), surgery and adjuvant radiotherapy in 0.5% (1/184), surgery and neoadjuvant radiotherapy in 0.5% (1/184), surgery and neoadjuvant radio and adjuvant chemotherapy in 0.5% (1/184), surgery and neoadjuvant chemo- and adjuvant radio-chemotherapy in 8% (15/184) or definitive chemotherapy in 1% (2/184)

^a^Cases: breast cancer (*n* = 4), cervix cancer (*n* = 1), prostate cancer (*n* = 2), colorectal cancer (*n* = 3), bladder cancer (*n* = 2), lung cancer (*n* = 2), renal cell carcinoma (*n* = 2), melanoma (*n* = 2), head and neck cancer (*n* = 1), thyroid carcinoma (*n* = 1)

**Table 2:** Clinico-pathological parameters and *SHOX2* and *SEPT9* methylation levels prior to treatment

Clinico-pathological parameters	Total number (*n*)	Methylation in plasma prior to treatment				
		Number (*n*)	Median *SEPT9* (%); IQR	Spearman’s *ρ, P*-value^a^	Median *SHOX2* (%); IQR	Spearman’s *ρ, P*-value^a^
All CRC cases	184 (100%)	155 (100%)				
Localisation						
Caecum	34 (18.5%)	33 (21.3%)	0.061; 0.37		0.072; 0.19	
Ascending colon	33 (17.9%)	30 (19.4%)	0.031; 0.25		0.036; 0.15	
Transverse colon	13 (7.1%)	8 (5.2%)	0.009; 0.08		0.009; 0.05	
Descending colon	10 (5.4%)	9 (5.8%)	0.155; 21.87		0.013; 7.99	
Sigmoid colon	44 (23.9%)	39 (25.2%)	0.055; 0.47		0.033; 0.12	
Rectum	43 (23.4%)	30 (19.4%)	0.161; 0.74	*P = *0.52	0.068; 0.25	*P = *0.40
Others^c^	7 (3.8%)	6 (3.9%)	0.616; 2.99		0.156; 0.35	
Primary tumour (*T*) category						
*T*_is_	1 (0.5%)	0 (0.0%)	N/A		N/A	
*T*_1_	14 (7.6%)	8 (5.2%)	0.000; 0.01		0.029; 0.10	
*T*_2_	30 (16.3%)	27 (17.4%)	0.016; 0.19		0.014; 0.09	
*T*_3_	103 (56.0%)	92 (59.3%)	0.123; 0.65	*ρ* = 0.28	0.044; 0.21	*ρ* = 0.25
*T*_4_	30 (16.3%)	24 (15.5%)	0.188; 0.80	*P < 0.001*	0.131; 0.21	*P = 0.002*
N/A^b^	6 (3.3%)	4 (2.6%)	0.413;0.58		0.023; 0.07	
Regional node (*N*) category						
*N*_0_	97 (52.7%)	89 (57.4%)	0.037; 0.25		0.032; 0.10	
*N*_1_	41 (22.3%)	36 (23.2%)	0.150; 0.59	*ρ = *0.28	0.071; 0.27	*ρ = *0.29
*N*_2_	27 (14.7%)	25 (16.1%)	0.393; 4.22	*P = 0.001*	0.171; 1.16	*P < 0.001*
*N*_*x*_	19 (10.3%)	5 (3.2%)	0.109; 0.63		0.000; 0.05	
Distant metastasis (*M*) category						
*M*_0_	159 (86.4%)	132 (85.2%)	0.049; 0.28		0.037; 0.14	
*M*_1a_	18 (9.8%)	16 (10.3%)	2.231; 4.18		0.296; 1.36	
*M*_1b_	7 (3.8%)	7 (4.5%)	0.393; 10.64	*P < 0.001*	0.121; 1.25	*P = 0.001*
Histopathological grade						
*G*_1_	9 (4.9%)	9 (5.8%)	0.000; 0.10		0.017; 0.05	
*G*_2_	134 (72.8%)	117 (75.5%)	0.055; 0.35	*ρ = *0.32	0.049; 0.14	*ρ = *0.26
*G*_3_	28 (15.2%)	23 (14.8%)	0.661; 4.45	*P < 0.001*	0.184; 1.28	*P = 0.001*
N/A^b^	13 (7.1%)	6 (3.9%)	0.074; 28.79		0.164; 13.89	
Lymphatic invasion (*L*)						
*L*_0_	121 (65.8%)	102 (65.8%)	0.041; 0.25		0.037; 0.12	
*L*_1_	53 (28.8%)	46 (29.7%)	0.215; 1.12	*P = 0.005*	0.092; 0.29	*P = 0.033*
N/A^b^	10 (5.4%)	7 (4.5%)	0.319; 24.01		0.228; 10.30	
Vascular invasion (*V*)						
*V*_0_	158 (85.9%)	134 (86.5%)	0.071; 0.53		0.043; 0.16	
*V*_1_	13 (7.1%)	11 (7.1%)	0.184; 0.39	*P = 0.86*	0.079; 0.18	*P = 0.20*
N/A^b^	13 (7.1%)	10 (6.4%)	0.090; 7.39		0.050; 3.18	
Surgical margin (*R*)						
*R*_0_	175 (95.1%)	148 (95.5%)	0.071; 0.47		0.049; 0.16	
*R*_1_	6 (3.3%)	4 (2.6%)	0.214; 1.61	*P = 0.66*	0.126; 0.74	*P = 0.38*
*R*_2_	0 (0.0%)	0 (0.0%)	N/A		N/A	
N/A^b^	3 (1.6%)	3 (1.9%)	24.036; N/A		10.301; N/A	
UICC stage						
I	37 (20.1%)	29 (18.7%)	0.000; 0.04		0.008; 0.08	
II	64 (34.8%)	58 (37.4%)	0.083; 0.31		0.034; 0.16	
III	48 (26.1%)	43 (27.7%)	0.125; 0.56	*ρ = *0.41	0.078; 0.22	*ρ = *0.32
IV	25 (13.6%)	23 (14.8%)	1.845; 4.34	*P < 0.001*	0.171; 1.25	*P < 0.001*
N/A^b^	10 (5.4%)	2 (1.3%)	0.104; N/A		0.023; N/A	
Extracapsular lymph node extension (ece)						
ece−/N0	129 (70.1%)	106 (68.4%)	0.041; 0.25		0.034; 0.13	
ece+	31 (16.8%)	26 (16.8%)	0.606; 3.24		0.143; 0.48	
N/A^b^	24 (13.0%)	23 (14.8%)	0.180; 1.82	*P = 0.001*	0.049; 0.80	*P = 0.016*

Clinico-pathological parameters of the CRC patient cohort (184 patients) and association with *SHOX2* and *SEPT9* plasma DNA methylation levels prior to treatment. Methylation levels prior to treatment were available for 155/184 patients.

^a^*P*-values refer to the following tests: Wilcoxon–Mann–Whitney *U* test (*R*_0_ vs. *R*_1,2_; *L*_0_ vs. *L*_1_; *V*_0_ vs. *V*_1_; *M*_0_ vs. *M*_1a,1b_; ece+ vs. ece−), Spearman’s rank correlation (T category, N category, UICC stage, G), ANOVA (tumour localisation),

^b^N/A data not available.

^c^Others (descending and sigmoid colon, rectosigmoid transition)

**Table 3:** Association of clinico-pathological parameters with SHOX2 and SEPT9 plasma DNA methylation levels after treatment

Clinico-pathological parameters	Methylation in plasma after treatment				
	Number (*n*)	Median *SEPT9* (%); IQR	Spearman’s *ρ, P*-value^a^	Median *SHOX2* [%]; IQR	Spearman’s *ρ, P*-value^a^
All CRC cases	108 (100%)				
Localisation					
Caecum	19 (17.6%)	0.008; 0.04		0.010; 0.03	
Ascending colon	20 (18.5%)	0.001; 0.03		0.006; 0.03	
Transverse colon	7 (6.5%)	0.000; 0.02		0.022; 0.06	
Descending colon	6 (5.6%)	0.003; 0.01		0.012; 0.01	
Sigmoid colon	32 (29.6%)	0.003; 0.03		0.014; 0.04	
Rectum	20 (18.5%)	0.000; 0.03	*P = *0.84	0.008; 0.02	*P = *0.56
Others^c^	4 (3.7%)	0.005; 0.03		0.055; 0.06	
Primary tumour (*T*) category					
*T*_is_	1 (0.9%)	N/A		N/A	
*T*_1_	8 (7.4%)	0.002; 0.03		0.007; 0.01	
*T*_2_	16 (14.8%)	0.000; 0.01		0.004; 0.03	
*T*_3_	60 (55.6%)	0.003; 0.02	*ρ = *0.13	0.010; 0.03	*ρ = *0.31
*T*_4_	20 (18.5%)	0.008; 0.04	*P = 0.20*	0.034; 0.07	*P = 0.001*
N/A^b^	3 (2.8%)	N/A		0.000; N/A	
Regional node (*N*) category					
*N*_0_	53 (49.1%)	0.001; 0.02		0.010; 0.03	
*N*_1_	25 (23.1%)	0.000; 0.01	*ρ = *0.088	0.016; 0.04	*ρ = *0.21
*N*_2_	16 (14.8%)	0.030; 2.24	*P = 0.40*	0.058; 0.26	*P = 0.047*
*N*_*x*_	14 (13.0%)	0.002; 0.02		0.006; 0.01	
Distant metastasis (*M*) category					
*M*_0_	97 (89.8%)	0.000; 0.01		0.009; 0.03	
*M*_1a_	9 (8.3%)	0.604; 20,22		0.115; 4.21	
*M*_1b_	2 (1.9%)	0.413; N/A	*P < 0.001*	0.156; N/A	*P < 0.001*
Histopathological grade					
*G*_1_	3 (2.8%)	0.004; N/A		0.013; N/A	
*G*_2_	80 (74.1%)	0.001; 0.03	*ρ = * −0.016	0.009; 0.04	*ρ = *0.11
*G*_3_	18 (16.7%)	0.004; 0.05	*P = 0.88*	0.020; 0.08	*P = 0.27*
N/A^b^	7 (6.5%)	0.003; 0.01		0.011; 0.02	
Lymphatic invasion (*L*)					
*L*_0_	68 (63.0%)	0.001; 0.01		0.009; 0.03	
*L*_1_	35 (32.4%)	0.003; 0.05	*P = 0.22*	0.018; 0.05	*P = 0.034*
N/A^b^	5 (4.6%)	0.000; 0.05		0.008; 0.01	
Vascular invasion (*V*)					
*V*_0_	96 (88.9%)	0.001; 0.02		0.010; 0.04	
*V*_1_	5 (4.6%)	0.009; 1.69	*P = 0.11*	0.020; 1.18	*P = 0.33*
N/A^b^	7 (6.5%)	0.000; 0.00		0.008; 0.01	
Surgical margin (*R*)					
*R*_0_	106 (98.1%)	0.001; 0.02		0.010; 0.03	
*R*_1_	2 (1.9%)	1.676; N/A	*P = 0.14*	1.159; N/A	*P = 0.73*
*R*_2_	0 (0.0%)	N/A		N/A	
N/A^b^	0 (0.0%)	N/A		N/A	
UICC stage					
I	19 (17.6%)	0.000; 0.01		0.008; 0.01	
II	38 (35.2%)	0.003; 0.02		0.011; 0.03	
III	31 (28.7%)	0.000; 0.01	*ρ = *0.27	0.012; 0.04	*ρ = *0.29
IV	11 (10.2%)	0.604; 3.19	*P = 0.008*	0.115; 2.30	*P = 0.003*
N/A^b^	9 (8.3%)	0.003; 0.03		0.007; 0.01	
Extracapsular lymph node extension (ece)					
ece−/N0	77 (71.3%)	0.001; 0.02		0.008; 0.02	
ece+	20 (18.5%)	0.013; 0.64		0.024; 0.17	
N/A^b^	11 (10.2%)	0.000; 0.01	*P = 0.023*	0.012; 0.03	*P = 0.012*

Methylation levels after treatment were available for 108/184 patients.

^a^*P*-values refer to the following tests: Wilcoxon−Mann−Whitney *U* test (*R*_0_ vs. *R*_1,2_; *L*_0_ vs. *L*_1_; *V*_0_ vs. *V*_1_; *M*_0_ vs. *M*_1a,1b_; ece+ vs. ece−), Spearman’s rank correlation (T category, N category, UICC stage, G), ANOVA (tumour localisation).

^b^N/A data not available.

^c^Others (descending and sigmoid colon, rectosigmoid transition)

Correspondingly, *SHOX2* methylation levels showed a gradual increment between UICC-stages (I-IV), nodal status (N_0_-N_2_), histological grades (G_1_–G_3_), and lymphatic invasion (L_0_-L_1_). No stepwise increase was recorded for local tumour categories (T_1_-T_4_) and distant metastasis (M_0_-M_1__b_, Tables 2 and 3). Sub-analysis of *SHOX2* methylation levels in distinct UICC stages showed a significant increase from stage III to IV (*P* = 0.036) but no significant elevation from stage I to II (*P* = 0.33) and stage II to III (*P* = 0.26, Fig. 2b).

Our previous study on *SHOX2* and *SEPT9* methylation for diagnosis, staging, prognosis, and monitoring of HNSCC patients included 224 cancer-free control patients.^15^ In this analysis, the set cut-offs for *SHOX2* (0.25%) and *SEPT9* (0.075%) methylation resulted in a specificity of 95%, and values below cut-off were considered sporadic background methylation levels known to occur in blood from healthy individuals and patients with benign diseases.^15^ Applying these previously validated cut-offs, a total of 78/155 (50.3%) cancer patients were *SEPT9*-positive, whereas only 32/155 (20.6%, *SHOX2*), 36/178 (20.2%, CEA), and 22/177 patients (12.4%, CA 19–9) showed levels above the specific cut-offs of the other analysed biomarkers. We recorded positive *SEPT9* methylation results in 4/29 (13.8%) UICC stage I, 29/58 (50.0%) stage II, 26/43 (60.5%) stage III, and 18/23 (78.3%) stage IV patients. Suspicious CEA levels were detected in 1/36 (2.8%) stage I, 13/62 (21.0%) stage II, 13/47 (27.7%) stage III, and 9/24 (37.5%) stage IV cases. *SHOX2* methylation and CA 19–9 levels above the cut-off were only traceable in 10/23 (43.5%) and 7/24 (29.2%) stage IV patients.

**Fig. 3:**
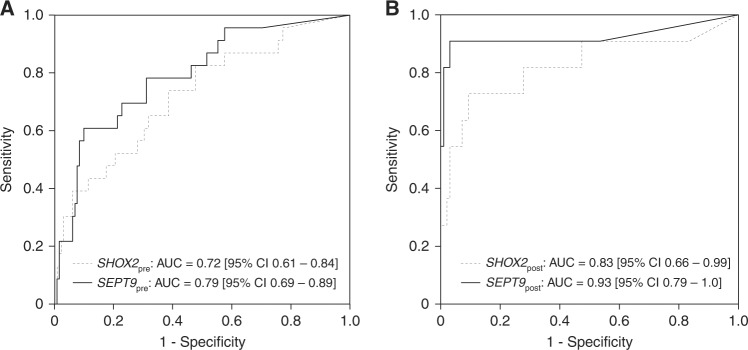
Detection of distant metastases—diagnostic accuracy. Receiver operating characteristic (ROC) of pre- (**a**) and post-therapeutic (**b**) ccfDNA methylation levels for the discrimination between metastasised (M_1a_, M_1b_; pre-therapeutic: *n* = 23 post-therapeutic: *n* = 11) and localised (M_0_; pre-therapeutic: *n* = 132, post-therapeutic: *n* = 97) colorectal cancers

Furthermore, *SEPT9* methylation showed the best ability to discriminate between localised and metastasised disease detecting 18/23 CRC with distant metastases in our cohort (78.3%, AUC = 0.79, [95% CI 0.69–0.89], Fig. 3a). In contrast, regarding the other biomarkers only 9/24 (37.5%, CEA, AUC = 0.73, [95% CI 0.64–0.83]), 10/23 (43.5%, *SHOX2*, AUC = 0.72, [95% CI 0.61–0.84]), and 7/24 (29.2%, CA 19–9, AUC = 0.64, [95% CI 0.51–0.78]) showed suspicious test results in M_1_ patients. *SEPT9* methylation also presented with the highest positivity rate in nodal-positive patients, although the capacity of all tested biomarkers was limited (*SEPT9:* 40/61 (65.6%); CEA: 21/66 (31.8%); *SHOX2*: 19/61 (31.1%); CA 19–9: 14/66 (21.2%)).

Both *SEPT9* and *SHOX2* showed a strong correlation with levels of the established tumour biomarkers CEA and CA 19–9 (*SEPT9*/CEA: Spearman’s *ρ* = 0.270, *P* = 0.001; *SEPT9*/CA 19–9: *ρ* = 0.161, *P* = 0.049; *SHOX2*/CEA: *ρ* = 0.313, *P* < 0.001; *SHOX2*/CA 19–9: *ρ* = 0.215, *P* = 0.008).

### CEA and CA 19-9 serum levels for CRC staging prior to treatment

Pre-therapeutic CEA and CA 19–9 serum levels were available for 178/184 (96.7%, CEA) and 177/184 patients (96.2%, CA 19–9), respectively. CEA showed a strong association with UICC stage, TNM, histological grade, extracapsular lymph node extension, vascular and lymphatic invasion (all *P* < 0.05) but not with tumour localisation (all *P* > 0.05, Table 4). In contrast, CA 19–9 expressed a significant relationship with UICC stage, nodal category, distant metastasis, histological grade, lymphatic invasion and extracapsular lymph node extension (all *P* < 0.05) but not with tumour localisation, T category and vascular invasion (all *P* > 0.05, Table 4).

**Table 4:** Clinico-pathological parameters and CEA and CA 19–9 serum levels prior to treatment

Clinico-pathological parameters	Total number (*n*)	CEA measurements (n)	Median CEA [%]; IQR	Spearman’s *ρ, P*-value^b^	CA19-9 measurements (n)	Median CA19-9 [%]; IQR	Spearman’s *ρ, P*-value^b^
All CRC cases	184 (100%)	178 (100%)			177 (100%)		
Localisation							
Caecum	34 (18.5%)	33 (18.5%)	1.600; 2.75		33 (18.6%)	11.90; 24.30	
Ascending colon	33 (17.9%)	32 (18.0%)	1.700; 3.25		32 (18.1%)	13.50; 19.33	
Transverse colon	13 (7.1%)	13 (7.3%)	1.800; 3.75		13 (7.3%)	10.00; 47.20	
Descending colon	10 (5.4%)	8 (4.5%)	2.900; 11.50		8 (4.5%)	11.30; 13.20	
Sigmoid colon	44 (23.9%)	43 (24.2%)	1.800; 1.50		42 (23.7%)	10.35; 12.18	
Rectum	43 (23.4%)	42 (23.6%)	1.900; 2.28	*P* = 0.43	42 (23.7%)	9.900; 8.98	*P* = 0.73
Othersc	7 (3.8%)	7 (3.9%)	8.200; 28.30		7 (4.0%)	8.100; 18.30	
Primary tumour (T) category							
T_is_	1 (0.5%)	1 (0.6%)	N/A		1 (0.6%)	N/A	
T_1_	14 (7.6%)	13 (7.3%)	1.000; 0.95		13 (7.3%)	7.500; 10.10	
T_2_	30 (16.3%)	28 (15.7%)	1.550; 1.68		27 (15.3%)	11.300; 8.60	
T_3_	103 (56.0%)	101 (56.7%)	1.900; 1.85	*ρ* = 0.39	101 (57.1%)	11.60; 13.20	*ρ* = 0.071
T_4_	30 (16.3%)	29 (16.3%)	7.700; 18.65	*P* = *0.001*	29 (16.4%)	9.900; 44.55	*P* = *0.35*
N/A^a^	6 (3.3%)	6 (3.4%)	1.550; 3.63		6 (3.4%)	6.500; 3.28	
Regional node (N) category							
N_0_	97 (52.7%)	95 (53.4%)	1.600; 1.80		94 (53.1%)	9.650; 11.45	
N_1_	41 (22.3%)	39 (21.9%)	2.100; 4.00	*ρ* = 0.33	39 (22.0%)	9.900; 12.40	*ρ* = 0.27
N_2_	27 (14.7%)	27 (15.2%)	2.900; 31.90	*P* = *0.001*	27 (15.3%)	26.70; 43,60	*P* = *0.001*
N_x_	19 (10.3%)	17 (9.6%)	1.500; 2.20		17 (9.6%)	8.600; 9.15	
Distant metastasis (M) category							
M_0_	159 (86.4%)	154 (86.5%)	1.700; 2.05		153 (86.4%)	10.00; 11.75	
M_1a_	18 (9.8%)	17 (9.6%)	2.600; 21.30	*P* = *0.001*	17 (9.6%)	19.80; 105.0	*P* = *0.026*
M_1b_	7 (3.8%)	7 (3.9%)	4.400; 36.20		7 (4.0%)	28.10; 32.90	
Histopathological grade							
G_1_	9 (4.9%)	8 (4.5%)	1.650; 2.13		8 (4.5%)	11.65; 10.75	
G_2_	134 (72.8%)	131 (73.6%)	1.800; 1.80	*ρ* = 0.22	130 (73.4%)	9.650; 12.38	*ρ* = 0.24
G_3_	28 (15.2%)	27 (15.2%)	2.900; 12.30	*P* = *0.004*	27 (15.3%)	17.70; 39.90	*P* = *0.002*
N/A^a^	13 (7.1%)	12 (6.7%)	1.700; 5.98		12 (6.8%)	9.950; 9.95	
Lymphatic invasion (L)							
L_0_	121 (65.8%)	116 (65.2%)	1.600; 1.80		115 (65.0%)	9.200; 11.30	
L_1_	53 (28.8%)	52 (29.2%)	2.550; 6.50	*P* = *0.001*	52 (29.4%)	17.05; 28.25	*P* = *0.001*
N/A^a^	10 (5.4%)	10 (5.6%)	2.000; 9.30		10 (5.6%)	9.700; 6.85	
Vascular invasion (V)							
V_0_	158 (85.9%)	153 (86.0%)	1.700; 2.00		152 (85.9%)	11.30; 14.58	
V_1_	13 (7.1%)	12 (6.7%)	5.05; 30.20	*P* = *0.031*	12 (6.8%)	12.00; 743.55	*P* = *0.49*
N/A^a^	13 (7.1%)	13 (7.3%)	2.100; 9.65		13 (7.3%)	9.500; 9.40	
Surgical margin (R)							
R_0_	175 (95.1%)	170 (95.5%)	1.800; 2.10		169 (95.5%)	11.30; 13.60	
R_1_	6 (3.3%)	5 (2.8%)	8.30; 129.20	*P* = *0.018*	5 (2.8%)	9.90; 2680.00	*P* = *0.91*
R_2_	0 (0.0%)	0 (0.0%)	N/A		0 (0.0%)	N/A	
N/A^a^	3 (1.6%)	3 (1.7%)	9.900; N/A		3 (1.7%)	6.200; N/A	
UICC stage							
I	37 (20.1%)	36 (20.2%)	1.350; 1.65		35 (19.8%)	10.00; 10.00	
II	64 (34.8%)	62 (34.8%)	1.750; 3.28		62 (35.0%)	9.050; 11.28	
III	48 (26.1%)	47 (26.4%)	2.300; 4,60	*ρ* = 0.39	47 (26.6%)	14.10; 20.20	*ρ* = 0.22
IV	25 (13.6%)	24 (13.5%)	2.850; 33.42	*P* = *0.001*	24 (13.6%)	19.90; 57.10	*P* = *0.005*
N/A^a^	10 (5.4%)	9 (5.1%)	0.900; 1.00		9 (5.1%)	8.600; 6.65	
Extracapsular lymph node extension (ece)							
ece-/N0	129 (70.1%)	123 (69.1%)	1.600; 1.80		122 (68.9%)	9.450; 11.25	
ece^d^	31 (16.8%)	31 (17.4%)	2.900; 28.30	*P* = *0.001*	31 (17.5%)	23.80; 41.50	*P* = *0.001*
N/A^a^	24 (13.0%)	24 (13.5%)	2.250; 6.25		24 (13.6%)	13.30; 10.10	

Clinico-pathological parameters of the CRC patient cohort (184 patients) and association with CEA and CA 19–9 serum levels. Serum levels prior to treatment were available for 178/184 patients (CEA) and 177/184 patients (CA 19–9)

^a^N/A: data not available

^b^*P*-values refer to the following tests: Wilcoxon–Mann–Whitney test (R_0_ vs. R_1,2_; L_0_ vs. L_1_; V_0_ vs. V_1_; M_0_ vs. M_1a,1b_; ece + vs. ece-), Spearman’s rank correlation (T category, N category, UICC stage, G), ANOVA (tumour localisation)

^c^Others (descending and sigmoid colon, rectosigmoid transition)

^d^Significant feature

Similar to the methylation biomarkers described above, median CEA blood levels rose from local to more invasive or systemic disease (UICC stage, TNM, histological grade, lymphatic and vascular invasion and extracapsular lymph node extension). Sub-analysis of CEA levels showed an increase from UICC stage I to II (*P* = 0.036), whereas no significant difference between stage II and III (*P* = 0.078) or stage III and IV could be detected (*P* = 0.12, Fig. 2c).

CA 19–9 also showed a gradual increase of median blood levels in relation to nodal category, distant metastasis, lymphatic and vascular invasion, and extracapsular lymph node extension but not for T category, UICC-stage, and histological grade (Table 4). Sub-analysis of CA 19–9 levels revealed a significant increase from UICC stage II to III (*P* = 0.033) but no higher median CA 19–9 level from stage I to II (*P* = 0.67) and stage III to IV (*P* = 0.30, Fig. 2d).

### *SHOX2* and *SEPT9* ccfDNA methylation in plasma after surgical treatment

**Fig. 4:**
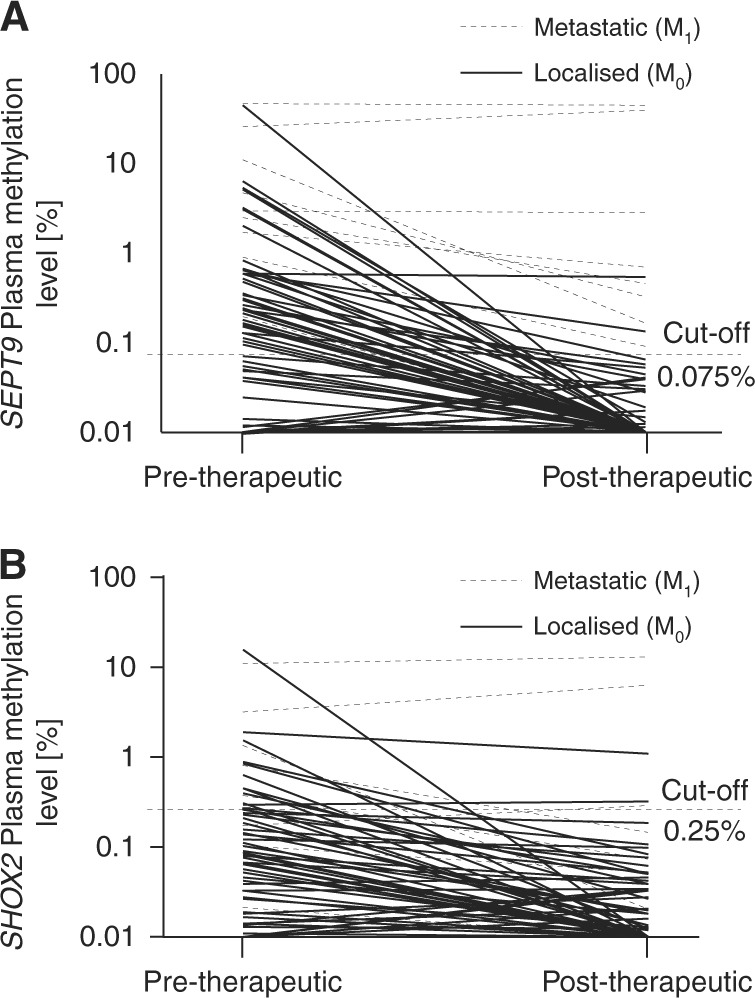
Pre and post-therapeutic ccfDNA methylation in matched samples from M_0_ and M_1_ CRC patients. Shown are pre and post-therapeutic *SEPT9* (**a**) and *SHOX2* (**b**) ccfDNA methylation levels in plasma of *n* = 9 M_1_ and *n* = 70 M_0_ patients with colorectal adenocarcinomas. Mean plasma ccfDNA methylation levels in M_0_ patients tend to decrease after therapy: *SEPT9*_pre-therapeutic_ = 1.16%, *SEPT9*_post-therapeutic_ = 0.019%, *P* = 0.089; *SHOX2*_pre-therapeutic_ = 0.38%, *SHOX2*_post-therapeutic_ = 0.040, *P* = 0.13. Mean plasma ccfDNA methylation levels in M_1_ patients remained high: *SEPT9*_pre-therapeutic_ = 10.32%, *SEPT9*_post-therapeutic_ = 9.41%, *P* = 0.67; *SHOX2*_pre-therapeutic_ = 1.85%, *SHOX2*_post-therapeutic_ = 2.15, *P* = 0.52. *P*-values refer to paired *t* test

**Fig. 5:**
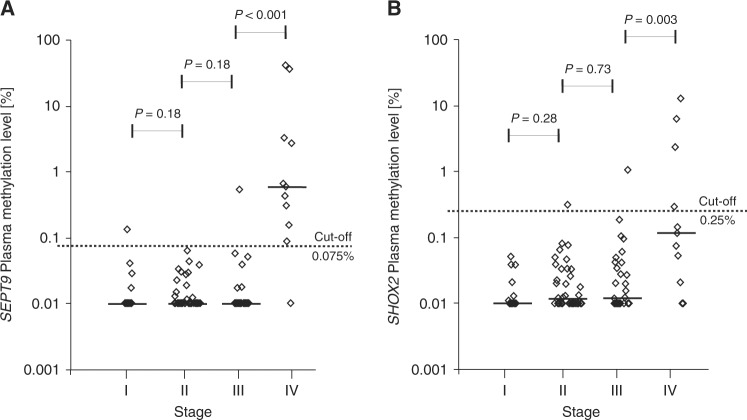
Stage-dependent post-therapeutic plasma ccfDNA methylation levels. *SEPT9* (**a**) and *SHOX2* (**b**) methylation in plasma ccfDNA of stage I (*n* = 19), II (*n* = 38), III (*n* = 31), and IV (*n* = 11) colorectal cancer patients 3–10 days after surgery. *P*-values refer to Wilcoxon Mann–Whitney *U* tests. Methylation levels below 0.01% were set to 0.01% in order to allow for a logarithmic illustration

Matched pre- and post-therapeutic cffDNA methylation results were available for 79 patients. In these patients, the mean total ccfDNA concentration in plasma quantified via the *ACTB* reference assay showed a significant 2.63-fold increase from 16.9 ng/3 mL to 44.5 ng/3 mL plasma after therapy (paired *t* test, *P* < 0.001). Post-therapeutic *SHOX2* and *SEPT9* ccfDNA methylation in matched samples from individual patients, however, showed a trend towards decreased levels in 70 patients with localised (M_0_, *SEPT9*: *P* = 0.089, *SHOX2*: *P* = 0.13) disease and no decrease in 9 patients with distant metastases (M_1_, *SEPT9*: *P* = 0.67, *SHOX2*: *P* = 0.52) (Fig. 4). The analysis of all patients enrolled, including unmatched patient samples, revealed that median *SEPT9* methylation levels dropped to barely traceable amounts 3–10 days after surgical tumour removal (Tables 2 and 3 and Fig. 5a, b). Patients with single (M_1__a_) and multiple distant metastases (M_1__b_) (M_0_: 3/97 (3.1 %) vs. M_1__a/b_: 10/11 (90.9%), *P* < 0.001), UICC stage IV (stage I: 18/19 (94.7%), stage II: 38/38 (100%) and stage III 30/31 (96.8%) vs. IV: 10/11 (90.9%), *P* < 0.001) and positive resection margins (R_1_), however, still showed post-therapeutic ccfDNA methylation positivity (Fig. 5a, b). *SHOX2* methylation levels were also elevated after resection in stage IV patients (4/11 (36.4%)) with a low positivity rate in all other stages (stage I: 0/19 (0%), stage II: 1/38 (2.6%) and stage III 1/31 (3.2%)) but compared to *SEPT9* methylation levels, only a small portion of M_1__a_/M_1__b_ patients (4/11, 36.4%) showed post-therapeutic *SHOX2*-positivity, while 2/97 (2.1%) M_0_ patients were *SHOX2*-positive. Consequently, post-therapeutic *SEPT9* ccfDNA methylation was shown to reliably discriminate between metastasised and localised disease with high diagnostic accuracy (AUC = 0.93 [95% CI 0.79–1.0], Fig. 3b).

## Discussion

Accurate staging of CRC using colonoscopy and up-to-date radiologic imaging is fundamental for treatment planning and prognosis. The periodically updated UICC and TNM staging system remains the worldwide standard for classification.^3^ Despite its significance, staging cancerous lesions solely on their radiologically determined anatomic extent neglects the emerging knowledge on the biological behavior and aggressiveness of solid tumours^28^ and also has considerable drawbacks in terms of accuracy, especially for the lymphatic invasion of the disease.^29,30^ This potential limitation has been highly debated in the most recent literature, particularly in the context of novel promising biomarkers.^31^ The combination of validated biomarkers with the established TNM system may therefore boost the efficiency of the existing regimens.

Here, we report that *SEPT9* and *SHOX2* ccfDNA hypermethylation performs outstandingly as an auxiliary molecular staging parameter. Especially the FDA approved blood-based methylation biomarker *SEPT9* was able to discriminate between pathological UICC and TNM stages in an incremental fashion and may therefore be able to provide an additional “molecular dimension” to the established staging system. Our results mirror the results of previous studies, which have shown lower plasma *SEPT9* methylation in earlier cancer stages compared to more advanced lesions.^32–34^ Above all, its ability to identify patients with a positive nodal status or distant metastases stresses the potential of *SEPT9* methylation as a biomarker adding valuable information to the TNM classification. This is even more important in the light of a fairly poor clinical lymph node staging.^29,30,35^ In contrast to patients with localised tumour stages, individuals with positive resection margins (R_1_) or distant metastases (M_1_) showed no decrease in *SEPT9* or *SHOX2* methylation after surgical resection, probably due to the residual tumour burden. These findings indicate that *SEPT9* ccfDNA methylation might be a potential biomarker for (occult) (micro-)metastases, advanced/systemic disease, or aggressive biological tumour behavior. With 25% of all potentially resectable liver metastases going undetected by standard imaging technique, high *SEPT9* and *SHOX2* methylation levels might indicate the need for extended imaging with MRI and/or PET-CT scans or an intraoperative ultrasound of the liver.^7,36^ Furthermore, blood-based biomarkers, e.g., ccfDNA methylation, offer the unique opportunity of gathering additional information on the extent of the disease prior to surgical treatment, especially as the final TNM classification relies on the resected specimen. In this situation, high *SEPT9* methylation levels might act as an additional biomarker to define high risk stage II patients, who currently are not eligible for adjuvant treatment but might indeed benefit from an intensified treatment.^4^ On the other hand, low *SEPT9* methylation levels might support the decision of postoperative watchful follow-up and might therefore avoid harmful adjuvant overtreatment. As previously reported for HNSCC patients,^15^ recurrence monitoring might be another potential application. Particularly, elevated *SEPT9* and *SHOX2* methylation levels after resection^37^ might suggest intensified monitoring and shortened follow-up for recurrence detection.

Hypermethylation of both tested gene loci has been associated with other cancer entities, carcinogenesis of which is influenced by alcohol and tobacco consumption, e.g., lung cancer, gastric cancer, cancer of the hepatobiliary tract system, pancreatic cancer, and head and neck cancer.^13,15,19,22,23^ According to the presumed non-specificity of *SEPT9* and *SHOX2* methylation levels regarding tumour-site and organ, they might also be applicable for the detection of occult second primary cancers.^15^

The application of serum protein biomarkers, i.e., CEA and CA 19–9, as biomarker for clinical CRC management has been intensively studied but found to lack sufficient sensitivity and specificity.^38^ While CEA performed particularly well in our study with regard to CRC staging when applying absolute values, even below the cut-off, the use of the accepted cut-off (5 ng/mL) diminished the power of CEA for staging considerably: Only 37.5% of patients with a systemic disease (UICC IV) showed CEA values above cut-off levels. Correspondingly, <60% of patients with a T_4_ tumour category had a CEA value above the level of 5 ng/mL. Although, CEA levels might add information to TNM staging as shown recently,^25,26,39^ it is still not included in the TNM system as prognostic biomarker. This neglect is partly reasoned by the varying distribution of protein biomarkers in CRC stages and by its interference with patients’ smoking status and comorbidities like metabolic syndrome.^40^ Therefore, some authors even suggest higher cut-off values.^41,42^ Accordingly, CA 19–9 accuracy is diminished in patients with cholestasis, pancreatitis, as well as individuals without Lewis antigen expression.^43^ Epigenetic biomarkers, on the other hand, are measured by different laboratory methods and might add valuable information to existing classification systems unaffected by common comorbidities. In addition to these advantages, blood based biomarkers tend to have a high acceptance among the population.^44,45^ Consequently, a myriad of other methylation biomarkers in tissue and plasma have been published.^46–51^ Pedersen et al.^49^ for example screened *BCAT1* and *IKZF1* methylation levels in over 2000 patients scheduled for colonoscopy. They reported a moderate sensitivity of 66% (85/129) for CRC detection and a stepwise increase for the positivity rate from stage I (38%) to stage IV (94%), which is in concordance with the gradual increase in more advanced tumours reported by us. Complementary, another group presented hypermethylation in several promoter regions—namely *SFRP1* and *SFRP2*, *SDC2* and *PRIMA1*—in CRC and adenomas.^51^ As previous described,^16^ hypermethylation seems to occur at a very early stage during CRC carcinogenesis and increases with progression. Very recently, Barault et al.^50^ analysed cancer-specific methylation patterns in 149 CRC cell lines and validated their panel in tumour tissue and plasma. They found at least one plasma biomarker in 85.7% of CRC samples with prognostic and diagnostic significance but excluded *SEPT9* from their analysis, since it failed to reach their stringent inclusion criteria. However, the FDA approved marker *SEPT9* remains the blood based biomarker with the highest level of validation, and other reported biomarkers still need additional prospectively validation.

In 2017, several meta-analysis^17,45,52^ showed a pooled sensitivity of 67 with 89% specificity for the *SEPT9* assay regarding the discrimination of CRC patients from individuals without a tumour. Although useful in diagnosis and screening, the authors do not suggest *SEPT9* as prognostic biomarker or therapeutic monitoring tool due to a lack of evidence. These findings stand in contrast to our results, which revealed *SEPT9* blood methylation levels to be highly correlated with one of the strongest prognostic parameters: the UICC stage. This might be explained by different experimental settings and study populations, especially in terms of an overrepresentation of metastatic patients in many of the previous reports. While further studies are warranted to support the prognostic value of *SEPT9* blood methylation, the general potential of methylation biomarkers as prognostic biomarker in CRC is evident. Garlan et al.^46^ for example reported that methylation biomarkers (*WIF1* and *NPY*) might act as prognostic biomarkers and stratify treatment responders into two groups with significantly differing outcomes.

No methylation biomarker published so far is able to detect or monitor CRC accurately enough to be used as stand-alone diagnostic tool. As a consequence, combination of promising biomarkers into a panel^53^ or combined with immunochemical Fecal Occult Blood Test (iFOBT) seems to present an attractive tool and might be necessary to reflect all molecular subtypes of CRC. A combination with iFOBT might further help to reduce problems that arise from the utility of a single blood-based methylation biomarker. The methylation biomarker performance is highly dependent on the sample quality, since inappropriate sample handling leads to the excessive release of DNA from lysing leucocytes^54–56^ resulting in a relative reduction of cancer-specific methylation biomarker levels. Furthermore, the quantification of tumourous ccfDNA in blood of a patient via methylation biomarkers is restricted to those genes that are hypermethylated in the individual patient’s tumour. Hence, intra and inter-tumoural methylation heterogeneity represents a general limitation of methylation biomarkers. Furthermore, other factors like age and time of blood collection seems to influence the *SEPT9* plasma levels and have to be taken into account. Herein, *SEPT9* shows a circadian rhythm^55^ which might impair sensitivity, especially in earlier lesions. Moreover, many genes undergo age-associated hypermethylation (reviewed by^57^) providing an explanation for higher *SEPT9* methylation levels in healthy individuals older than 60 years compared to their younger counterparts.^32^ Additionally, the time point of post-surgical blood collection might also have a critical impact on the reliability of our results due to an increased level of total ccfDNA based on healing processes or undegraded remnant tumour ccfDNA. However, the estimated half-life of ccfDNA has been reported to be a matter of minutes to hours^58,59^ suggesting that blood sampling three days after surgery might be appropriate.

Designed in a prospective manner, our study lacks several typical drawbacks, e.g., missing data, selection, and information bias. Nonetheless, we are aware of certain limitations. We were not able to follow enough patients for a profound survival analysis and were therefore not able to generate analyses of patients’ outcome.

Our results further highlight that methylation biomarkers alone have limited use for CRC screening owing to low biomarker levels in blood from patients with early stage cancers compared to advanced tumour stages. Consequently, small and clinically occult tumours that would have the highest chance of cure would very likely go undetected. The significant association of *SEPT9* and *SHOX2* methylation and TNM categories, a strong prognostic indicator of survival,^1^ however, highlights the potential of blood-based biomarkers.

In conclusion, methylation testing in plasma is a powerful additional diagnostic tool that, together with the recent TNM classification, facilitates molecular disease staging of CRC. Patients with initially high biomarker levels might benefit from intensified treatment and close post-therapeutic surveillance. The early detection of recurrent/metastatic disease could lead to earlier consecutive treatment, thereby improving patients’ outcomes. Post-therapeutically elevated ccfDNA methylation levels appear to indicate the presence of residual disease and distant metastases. These patients might benefit from an early initiation of a systemic treatment.

## Electronic supplementary material

Supplemental Figure 1

Supplemental Table 1
